# Impact of the Method Used to Select Gas Exchange Data for Estimating the Resting Metabolic Rate, as Supplied by Breath-by-Breath Metabolic Carts

**DOI:** 10.3390/nu12020487

**Published:** 2020-02-14

**Authors:** Juan M.A. Alcantara, Guillermo Sanchez-Delgado, Francisco J. Amaro-Gahete, Jose E. Galgani, Jonatan R. Ruiz

**Affiliations:** 1 PROFITH “PROmoting FITness and Health Through Physical Activity” Research Group, Sport and Health University Research Institute (iMUDS), Department of Physical and Sports Education, Faculty of Sport Sciences, University of Granada, 18011 Granada, Spain; gsanchezdelgado@ugr.es (G.S.-D.); amarof@ugr.es (F.J.A.-G.); ruizj@ugr.es (J.R.R.); 2 Pennington Biomedical Research Center, Baton Rouge, LA 70808, USA; 3 EFFECTS-262 Research Group, Department of Physiology, School of Medicine, University of Granada, 18011 Granada, Spain; 4 Departamento de Ciencias de la Salud, Carrera de Nutrición y Dietética, Facultad de Medicina, Pontificia Universidad Católica de Chile, Santiago, Chile; Departamento de Nutrición, Diabetes y Metabolismo, Facultad de Medicina, Universidad Pontificia Católica de Chile, 8330023 Santiago, Chile.; jgalgani@uc.cl

**Keywords:** resting energy expenditure, CCM express, CPX Ultima CardiO2, indirect calorimetry, macronutrient oxidation

## Abstract

The method used to select representative gas exchange data from large datasets influences the resting metabolic rate (RMR) returned. This study determines which of three methods yields the lowest RMR (as recommended for use in human energy balance studies), and in which method the greatest variance in RMR is explained by classical determinants of this variable. A total of 107 young and 74 middle-aged adults underwent a 30 min RMR examination using a breath-by-breath metabolic cart. Three gas exchange data selection methods were used: (i) steady state (SSt) for 3, 4, 5, or 10 min, (ii) a pre-defined time interval (TI), i.e., 6–10, 11–15, 16–20, 21–25, 26–30, 6–25, or 6–30 min, and (iii) “filtering”, setting thresholds depending on the mean RMR value obtained. In both cohorts, the RMRs yielded by the SSt and filtering methods were significantly lower (*p* < 0.021) than those yielded by the TI method. No differences in RMR were seen under the different conditions of the SSt method, or of the filtering method. No differences were seen between the methods in terms of the variance in RMR explained by its classical determinants. In conclusion, the SSt and filtering methods return the lowest RMRs and intra-measurement coefficients of variation when using breath-by-breath metabolic carts.

## 1. Introduction

The resting metabolic rate (RMR) is the lowest energy expenditure of a person who is awake [1], after at least 12 h of fasting, being in physical rest, and in a state of mental relaxation in an ambient environmental temperature; it accounts for some 60–70% of the total daily energy expenditure [2]. The assessment of RMR is important when studying human energy balance, both in clinical and research settings [2,3]. Indirect calorimetry is the reference method for assessing RMR [2,4,5,6], which is estimated from the consumption of oxygen (VO_2_) and the production of carbon dioxide (VCO_2_) [6]. The measurement of VO_2_ and VCO_2_, together with urinary nitrogen excretion, also allows for the estimation of the nutrient (carbohydrate and fat) oxidation rate [7]. Indeed, the VCO_2_/VO_2_ ratio, i.e., the respiratory quotient (RQ), is an indicator of the relative predominance of fat (FATOx) and carbohydrate (CHOOx) oxidation.

The assessment of RMR using indirect calorimetry is normally performed over a 10–30 min period. It is widely assumed that the first 5 min of data recorded should be discarded [8,9]. A short (e.g., 5 min) steady respiratory state period, i.e., a period in which the indirect calorimetry record is markedly stable, then has to be selected from the remaining dataset for estimating the RMR [6,7,10]. The assumption that steady state (SSt) methods for gas exchange data selection provide a better estimate of RMR than the other methods available arose from studies performed in ventilated patients [10]. However, there is no strong evidence that the same can be assumed in healthy, non-ventilated subjects—and indeed different methods have been used. These methods can be grouped into three categories: (i) the selection of an SSt (defined as that providing a coefficient of variance [CV] of <10% for VO_2_, VCO_2_, and minute ventilation [VE], and of <5% for RQ [11]), (ii) the selection of a pre-defined time interval (TI), without taking the stability of the results obtained into consideration [11], and (iii) “filtering”, in which data above or below a given RMR threshold are discarded. Both the SSt and TI methods can be used under different time conditions [1]. Unfortunately, the use of different methods for gas exchange data selection could result in different estimates of RMR and nutrient oxidation rates being made [1,11,12]. For instance, in a study involving healthy subjects, Irving et al. [1] reported RMR estimates made by the SSt and TI methods to differ by some −101 to +121 kcal/day.

Certainly, SSt-based RMR estimates are usually lower than those provided by the TI method [1,11]. Given the above definition of RMR [1], it has been proposed that the lowest estimates obtained by the SSt method should be deemed more accurate than those provided by TI. However, the under-estimation of the homeostatic RMR cannot be ruled out in the SSt method, nor has any study checked whether the filtering methods for data selection provide lower RMR estimates than either SSt or TI.

The present work examines whether the SSt, TI, or filtering method yields the lowest RMR value in healthy, non-ventilated subjects, and determines in which method the greatest variance in RMR is explained by the classical determinants of this variable (i.e., body weight, body composition, and sex) [13].

## 2. Materials and Methods

### 2.1. Study Subjects

The participants of this retrospective study were 107 young adults (72 women) enrolled in the ACTIBATE study [14] and 74 middle-aged adults (39 women) enrolled in the FIT-AGEING study [15]. Detailed information about the methodology of the aforementioned studies can be found elsewhere [14,15]. Briefly, the inclusion criteria were: (i) being physically inactive (<20 min of moderate–vigorous physical activity on <3 days/week), (ii) having a stable body weight (change <3 kg over the last 3 months [ACTIBATE] or <5 kg over the last 5 months [FIT-AGEING]), (iii) not being enrolled in a weight loss program, (iv) not being a smoker, (v) not suffering from an acute or chronic illness, and (vi) not being pregnant. The ACTIBATE study protocol was approved by the Committee for Research Involving Human Subjects at the University of Granada (Reference #924) and the *Servicio Andaluz de Salud (Centro de Granada, CEI-Granada*), while the FIT-AGEING was approved by the Human Research Ethics Committee of the *Junta de Andalucía* (0838-N-2017). Both studies were performed in accordance with the Declaration of Helsinki (2013 revision) and registered on the clinicaltrials.gov platform (IDs: NCT02365129 for the ACTIBATE study and NCT03334357 for the FIT-AGEING study). Oral and written informed consent was obtained from all the subjects before their enrolment.

### 2.2. Procedures

In both the above studies, subjects underwent a 30 min indirect calorimetry assessment of RMR at rest, early in the morning, following an overnight fast. All subjects were instructed to refrain from moderate (24 h) and vigorous physical activity (48 h) before the test day. On the previous evening, all subjects consumed a standardized meal of an egg omelet, boiled rice, and tomato sauce (ad libitum amounts). They were also instructed to avoid physical activity after they woke up, and to come to the research center by car or bus early in the morning, having had no breakfast (ensuring a ~12 h fast). Upon arrival, and after confirming their compliance with these above conditions, body weight and height were measured using a Seca model 799 electronic column scale (seca GmbH & Co. KG, Hamburg, Germany) with subjects barefoot and wearing light clothing. Thereafter, the subjects laid on a bed in the supine position for 20–30 min. Gas exchange data were then recorded by indirect calorimetry for 30 min in a quiet room with dim lighting, controlled at 22–24 °C and 35–45% relative humidity [8]. During this time the subjects were covered with a bed sheet and instructed to remain silent, stay awake, avoid fidgeting, and to breathe normally.

### 2.3. Gas Exchange Assessments

Gas exchange was recorded using either a CCM Express or a CPX Ultima CardiO2 (two different devices were used only in the middle-aged adults cohort) breath-by-breath metabolic cart (Medical Graphics Corp, St. Paul, MN, USA). Both instruments require the use of a face mask equipped with a Directconnect™ flow sensor (Medical Graphics Corp, St. Paul, MN, USA). Both determine VCO_2_ using a non-dispersive infrared analyzer, and both determine VO_2_ using a galvanic fuel cell [16]. The flow rate was calibrated using a 3 L syringe at the beginning of every test. The gas analyzers were calibrated before each measurement using standard gases according to the manufacturers’ instructions [16].

### 2.4. Methods for Gas Exchange Data Selection

The collected gas exchange data were processed using MGCDiagnostic^®^ Breeze Suite 8.1.0.54 SP7 software (Medical Graphics Corp., St. Paul, MN, USA) to yield a data point for each variable for every minute (i.e., the means of all ventilation data (per minute ventilation data—*p*MVD) for each particular minute). The first 5 min of data were discarded [8,9]; the remaining 25 min period dataset was processed using three different methods to select representative gas exchange data for determining the RMR and nutrient oxidation rate.

#### 2.4.1. Time Interval Method

Short TIs of 6–10 min, 11–15 min, 16–20 min, 21–25 min, and 26–30 min, and long TIs of 6–25 min and 6–30 min were established [11], and the means of the *p*MVD values for all variables available for these time periods calculated. These processed data were used to calculate the RMR and nutrient oxidation rate (see below for details).

#### 2.4.2. Steady-State Time Method

The CVs of VO_2_, VCO_2_, VE, and RQ were calculated for every period of 3, 4, 5, and 10 min (e.g., for the 3 min SSt we processed all the 25 min period datasets and we examined the 6th to 8th min period, the 7th to 9th period, etc.) and the mean CVs for each variable calculated for each time period. The periods selected for the final analyses were those with the lowest mean CV for each (e.g., from the 3 min SSt examined periods, we selected the 7th to 9th) [11]. The means of the available *p*MVD values for these time periods were then calculated. These processed data were used to calculate the RMR and nutrient oxidation rate (see below for details).

#### 2.4.3. Filtering Method

The *p*MVD values for VO_2_ and VCO_2_ for the entire 25 min data collection period—i.e., with no division into SSt or TI periods—were used to calculate the mean_25 min_ RMR (see below for details). Furthermore, *p*MVD RMR values were also calculated, discarding either (i) those values <85% or >115% of the mean_25 min_ RMR (low filter), (ii) <90% or >110% of the mean_25 min_ RMR (medium filter), or (iii) <95% or >105% of the mean_25 min_ RMR (strong filter). For the minutes that passed these filters, the means were calculated for all *p*MVD values available.

### 2.5. Calculating the Resting Metabolic and Nutrient Oxidation Rates

Weir’s equation (assuming zero urinary nitrogen excretion) [17] was used to calculate RMR values from the mean *p*MVD for VO_2_ and VCO_2_ obtained with each gas exchange data selection method. The FATOx and CHOOx rates were calculated using Frayn’s stoichiometric equations [18], also assuming zero urinary nitrogen excretion. Finally, the mean RMR, RQ, VO_2_, VCO_2_, FATOx, and CHOOx were calculated for all gas exchange data selection methods under each different condition.

### 2.6. Body Composition

Body composition was determined by dual energy X-ray absorptiometry using a Discovery Wi device (Hologic, Inc., Bedford, MA, USA). Quality controls, the positioning of participants and analysis of the results were performed according to the manufacturer’s recommendations.

### 2.7. Statistical analysis

Results are presented as mean±SD unless otherwise stated. All analyses were conducted using the Statistical Package for the Social Sciences v.22.0 (IBM SPSS Statistics, IBM Corporation, Chicago, IL, USA). Significance was set at *p* < 0.05. Repeated-measures analysis of variance (ANOVA) with a post-hoc Bonferroni test was used to detect differences in RMR, RQ, VO_2_, VCO_2_, FATOx, and CHOOx estimates across the methods for gas exchange data selection. The CVs for VO_2_, VCO_2_, RQ, and VE obtained via the different methods were also compared. Two different ANOVA models were used: one with four levels of fixed factors (i.e., short TI, long TI, SSt, and filtering), and one with 14 levels (all methods and their different conditions).

The differences between the methods in terms of the variance in RMR explained by its classical determinants (i.e., body weight, body composition [lean and fat masses], and sex) [13] were examined by either simple linear regression (associations between RMR and body weight) or multiple linear regression (associations between RMR and sex and body weight; RMR and sex and lean and fat masses).

## 3. Results

Table 1 provides descriptive data for the subjects in both cohorts.

### 3.1. Influence of the Gas Exchange Data Selection Method on Estimates of RMR, RQ and Nutrient Oxidation

**Figure 1:**
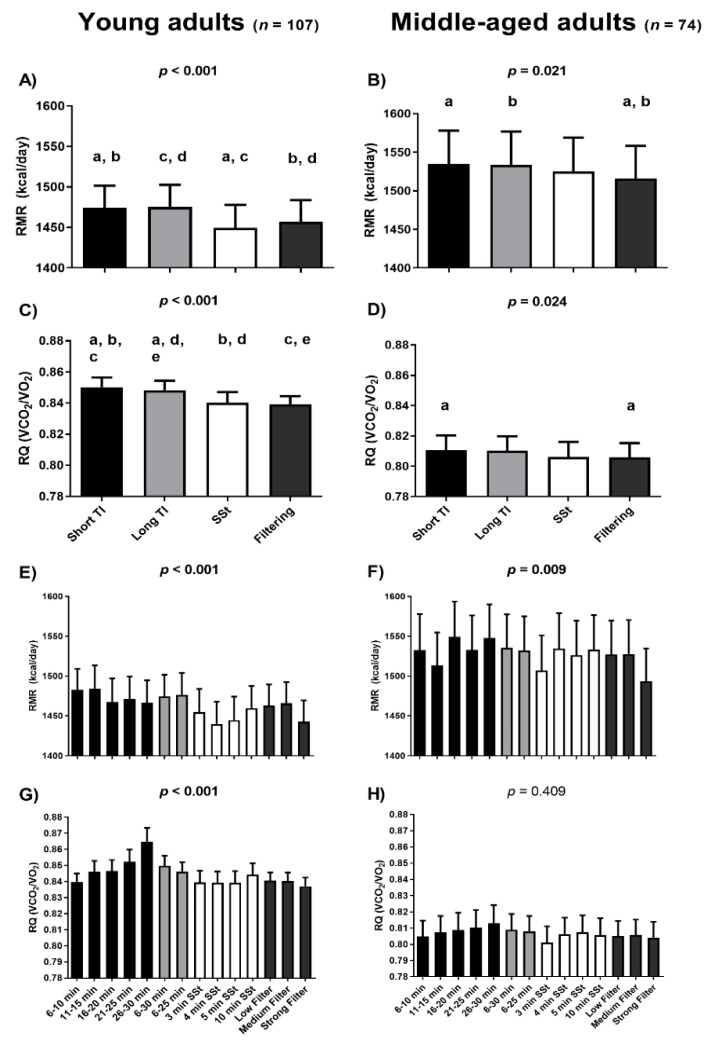
Differences among gas exchange data selection methods with respect to resting metabolic rate (RMR) and respiratory quotient (RQ) estimates. Black columns represent short time interval (TI) periods (i.e., the means of the per minute ventilation data (*p*MVD]) values for all variables available for these time periods, panels **A**–**D**; the *p*MVD values for each short TI period, panels **E**,**F**). Light grey columns represent long TI periods (i.e., the means of the *p*MVD values for all variables available for these time periods, panels **A**–**D**; the means of the *p*MVD values for each long TI period, panels **E,F**). White columns represent steady state (SSt) periods (i.e., the means of the *p*MVD values for all variables available for these SSt periods, panels **A**–**D**; the means of the *p*MVD values for each SSt period, panels **E**,**F**). Dark grey columns represent filtering methods (i.e., the means of the *p*MVD values for all variables available for these filtering periods, panels **A**–**D**; the means of the *p*MVD values for each filtering period, panels **E**,**F**). *p*-values come from repeated-measures analysis of variance (ANOVA). Identical indicatory letters highlight differences as determined by post-hoc Bonferroni analysis. Data are presented as mean and standard error of the mean (SEM). Min: minutes; VCO_2_: production of carbon dioxide; VO_2_: consumption of oxygen.

Figure 1 shows the RMR and RQ estimates yielded by the gas exchange data selection methods for both the young and middle-aged adults. In the young adults, the short and long TI methods provided higher mean RMR estimates than either the SSt or filtering methods (taking all conditions together; post-hoc Bonferroni *p* < 0.001; Figure 1A). In the middle-aged adults they also provided higher mean RMR estimates than the filtering method (taking all conditions together; post-hoc Bonferroni *p* < 0.001; Figure 1B). No differences were seen between the RMR estimates yielded by the short and long TIs, nor between the SSt and the filtering methods (taking all conditions together) in either the young or the middle-aged adults (all post-hoc Bonferroni *p* = 1.000; Figure 1A,B). For the young adults, the lowest mean RMR values were obtained with the SSt 4 min method (1440 kcal/day; Figure 1E); however, the SSt 4 min method was only statistically different from the TI 6–10 min and the TI 11–15 min. In the middle-aged adults the lowest mean RMR values were provided by the strong-filter method (1493 kcal/day; Figure 1F); the strong-filter method was statistically different from all the different methods, with the exception of the TI 11–15 min, and the SSt 3, 4, and 5 min conditions. Table S1 shows the comparisons (i.e., post-hoc Bonferroni) between the different methods. Similar patterns were observed when analyzing the influence of gas exchange data selection method on VO_2_ and VCO_2_ estimates (Figure S1).

Lastly, the periods in which the SSt were achieved (with the different SSt methods applied) in the young and the middle-aged adults are presented in Figure S2. We observed that ~50% of young and middle-aged adults achieved their SSts (i.e., the one presenting lower mean CV) during the first half of the 30 min indirect calorimetry assessment. On the other hand, we found differences between the first steady state achieved (i.e., the first period in which the CVs of VO_2_ < 10, VCO_2_ < 10, VE < 10, and RQ < 5) and the “best” SSt achieved (i.e., the period with the lowest mean CVs) in RMR estimation in young adults (Figure S3).

**Figure 2:**
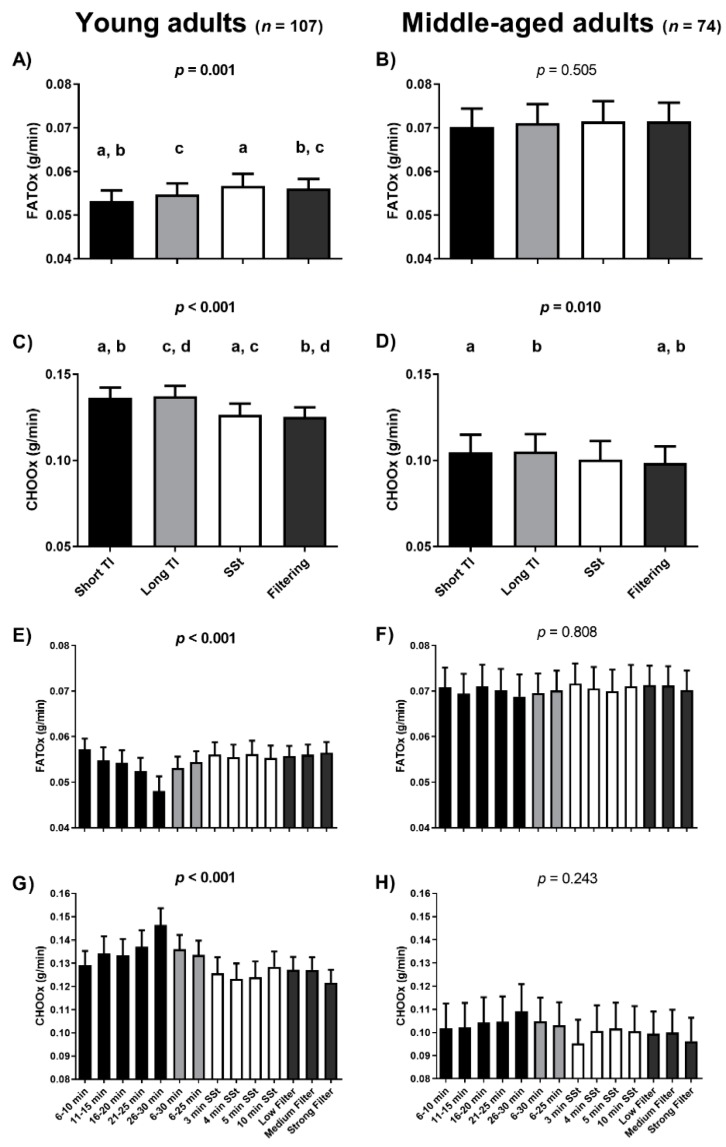
Differences among gas exchange data selection methods with respect to fat oxidation (FATOx) and carbohydrate oxidation (CHOOx) rates. Black columns represent short time interval (TI) periods (i.e., the means of the per minute ventilation data [*p*MVD] values for all variables available for these time periods, panels **A**–**D**; the *p*MVD values for each short TI period, panels **E**,**F**). Light grey columns represent long TI periods (i.e., the means of the *p*MVD values for all variables available for these time periods, panels **A**–**D**; the means of the *p*MVD values for each long TI period, panels **E**,**F**). White columns represent steady state (SSt) periods (i.e., the means of the *p*MVD values for all variables available for these SSt periods, panels **A**–**D**; the means of the *p*MVD values for each SSt period, panels **E**,**F**). Dark grey columns represent filtering methods (i.e., the means of the *p*MVD values for all variables available for these filtering periods, panels **A**–**D**; the means of the *p*MVD values for each filtering period, panels **E**,**F**). *p*-values come from repeated-measures analysis of variance (ANOVA). Identical indicatory letters highlight differences as determined by post-hoc Bonferroni analysis. Data are presented as mean and standard error of the mean (SEM). Min: minutes.

The RQ estimates yielded by the short TI method were significantly higher than all others in the young adult cohort (all post-hoc Bonferroni *p* < 0.002; Figure 1C) and that filtering method (taking all conditions together) in the middle-aged adults cohort (post-hoc Bonferroni *p* = 0.038; Figure 1D). Moreover, the long TI method provided higher RQ estimates than the SSt and filtering methods (taking all conditions together) in the young adult (both Bonferroni post-hoc *p* < 0.013; Figure 1C). No differences in RQ estimates were seen when comparing the SSt and filtering methods (taking all conditions together) in either the young or the middle-aged adults (post-hoc Bonferroni *p* = 1.000; Figure 1C,D). Furthermore, the long TI and the SSt were not significantly different than either the short TI method or the filtering methods (taking all conditions together) in the middle-aged adults (Figure 1D). The lowest mean RQ values were obtained when using the strong-filter method (0.84) in the young adults (Figure 1G). However, the strong-filter method was only statistically different from the TI 26–30 min and the TI 6–30 min. The lowest mean RQ values were obtained when using the SSt 3 min method (0.80) in the middle-aged adults (Figure 1H). However, no statistical differences were observed. As expected, the data selection methods yielding higher RQ estimates also provided higher CHOOx and lower FATOx estimates, and vice versa (Figure 2).

### 3.2. Differences between the Methods in Terms of the Variance in RMR Explained by Its Classical Determinants

The variance in RMR explained by body weight (taking all conditions together) was 36%, 36%, 34% and 38% for the short TI, long TI, SSt, and filtering methods respectively in young adults, and 50%, 51%, 52% and 51% respectively in the middle-aged adults. The most explained variance was obtained using the low-filter method (40%) in young adults and the TI 6–10 min method (54%) in the middle-aged adults.

The variance explained increased to 34–45% and 54–68% in the young and middle-aged adults respectively after including subject sex in the model (Table 2). The most explained variance was obtained with the low-filter method in young adults, and both the TI 21–25 min and the medium-filter methods in the middle-aged adults. However, little difference was seen among the methods in terms of the variance explained by the classical determinants of RMR (Table 2).

A further model including subject sex, lean, and fat masses increased the variance in RMR explained by ~5% in the young adults, but not in the middle-aged adults (Table 3). The low-filter method explained the greatest variance in RMR in the young adults, and the TI 21–25 min method did so in the middle-aged adults. However, once again, little difference was seen among the methods in terms of the variance explained by the classical determinants of RMR (Table 3).

## 4. Discussion

The present results show that when using breath-by-breath metabolic carts, RMR, and RQ estimates yielded by the SSt and filtering methods are lower than those yielded by the TI method, while no differences were seen between the SSt and filtering methods. The variance in the RMR explained by its classical determinants (i.e., weight, body composition, and sex) was similar in all methods. These findings largely concurred in the cohorts of young adults and middle-aged adults examined, which further reinforced the consistency of the results.

### 4.1. Influence of the Gas Exchange Data Selection Method on RMR, RQ and Nutrient Oxidation

Given that RMR is defined as the lowest energy expenditure of a person who is awake [1], the present results suggest that the SSt and filtering methods provide better RMR estimates. These results are in line with those reported in the literature [1,12] and concur with those reported in our previous study [11], in which lower RMR and RQ estimates were yielded by the SSt method than the TI method. As expected, the SSt method showed less variability in the results returned than the TI method (Figures S4 and S5). This supports the notion that the assessment of stable state respiratory gas exchange variables provides the best results [8,19].

The SSt method purportedly reflects the baseline physiological state [10], thus reflecting the homeostatic RMR and nutrient oxidation rates [20,21]. Reeves et al. [19] reported that SSt measurements for assessing RMR were more accurate when taking data over short periods (e.g., 30 min). In line with this, McClave et al. [10] reported that the RMR obtained with the SSt 5 min method provides an accurate representation of the 24 h total energy expenditure in bedridden hospitalized patients owing to their low level of physical activity. However, in healthy individuals, the RMR does not represent the 24 h total energy expenditure [6]. McClave et al. [10] recommended that: (i) a steady respiratory state should be considered reached when changes in the CV of VO_2_ and VCO_2_ are <10% over a period of 5 consecutive min, (ii) RMR assessment should end when a steady respiratory state is achieved, and (iii) when a steady respiratory state cannot be reached, a more prolonged test (≥60 min) becomes necessary. Reeves et al. [19] showed that reducing the steady respiratory state time period for data collection from 5 to 4 min resulted in acceptable RMR values. It has been suggested that reducing it to 3 min might underestimate the RMR [19], but in our previous study [11] no differences were seen between the RMRs provided by the SSt 3, 4, or 5 min conditions. The present results agree with our previous findings in a smaller sample of young adults (17 vs. the present 107) and middle-aged adults [11]. In addition, the present results for both independent cohorts were similar (i.e., replicated). As we mentioned previously, the assessment of RMR using indirect calorimetry is normally performed over a 30 min period, and our results suggest that the SSt becomes more stable (i.e., less variable) as the RMR measurement progresses (Figure S3). On the other hand, we observed that after ~15 min of measurement, ~50% of the young and the middle-aged adults achieved the SSt (Figure S2). Thus, based on our results we would recommend that when RMR is assessed using indirect calorimetry the measurement should last at least 30 min, to obtain the most stable results as possible reducing the variability (Figure S3). Moreover, it could be interesting to test whether the stability increases (or not) if the RMR measurement lasts more than 30 min.

To our knowledge, no other study has compared the TI, SSt, and filtering methods, rendering further comparisons impossible. However, the present results suggest that those provided by the filtering method are similar to those provided by the SSt method. It might therefore be used as an alternative in subjects in whom a steady respiratory state is not achieved (although the present subjects all reached a steady respiratory state, which might bias this suggestion).

Mean FATOx was higher and CHOOx lower, when determined via the SSt and filtering methods compared to the TI method, with no differences seen among the different SSt periods or among the different filtering conditions. In contrast, differences were observed among the TI periods in terms of both the RQ and nutrient oxidation rates, with an increase in the RQ seen over each test period (more pronounced in the young adult cohort—Figure 2). This gradual increase in the RQ, which might influence the nutrient oxidation rates estimates, may be related to the metabolic cart rather than subject factors or the method of gas exchange data selection used, since this increase was also observed in a previous study involving different subjects but using the same metabolic carts [11].

### 4.2. Differences between the Methods in Terms of the Variance in RMR Explained by Its Classical Determinants

For the middle-aged adults, the variance in RMR explained by its classical determinants across the gas exchange data selection methods was in line with the results of previous studies (36–56% explained by body weight [3,22,23,24]), although in the young adult cohort the variance explained was less than in the majority of the aforementioned studies. When comparing with previous studies in which body composition was included in the regression model, the variance in RMR explained across methods in the present study was also lower. In fact, Müller et al. [25] reported 72% of the variance in RMR to be explained by sex, lean mass, and fat mass. Korth et al. [22] reported 75% of the variance to be explained by lean mass alone, while Mifflin et al. [23] reported a value of 64%. Galgani and Castro-Sepulveda [3] reported 75% of the variance in RMR to be explained by fat-free mass, fat mass, and age. In the present study, the variance explained for the middle-aged adults was in line with the aforementioned studies (55–68% depending on the gas exchange data selection method used—Table 3). The differences across studies might be related, to a greater or lesser extent, to the accuracy of the metabolic cart [16]. In fact, in that study [16] we compared the inter-day reliability and the congruent validity of the CCM Express and the CPX Ultima CardiO2. Firstly, we observed that the CCM Express metabolic cart is more reliable than the CPX Ultima CardiO2 (i.e., less RMR inter-day differences: 158 ± 154 kcal/day vs. 219 ± 185 kcal/day for the CCM Express and the CPX Ultima CardiO2 respectively) [16]. Secondly, we observed that the RMR values obtained using the CPX Ultima CardiO2 were higher than the values obtained using the CCM Express metabolic cart (mean difference between metabolic carts of 65 ± 161 kcal/day on study day 1 and 94 ± 161 kcal/day on study day 2) [16]. Thus, as mentioned, the differences across studies could be related to the accuracy of the metabolic cart, and it may be that neither of the breath-by-breath carts used (i.e., the CCM Express and CPX Ultima CardiO2) is sufficiently accurate for measuring RMR if the ability to explain the variance in RMR is taken as an indirect indicator of accuracy. However, the variance explained was quite similar for all the gas exchange data selection methods used; it may not, therefore, be “method-dependent”.

The present results should be considered with some caution. The assessment of RMR was performed using two different breath-by-breath metabolic carts indiscriminately, both of which were equipped with a face-mask. The use of other metabolic carts or other gas collection systems (e.g., canopy collection) might influence the RMR results obtained [26,27]. Further studies involving different metabolic carts and gas collection systems, as well as with different subject populations, are needed to confirm the results.

## 5. Conclusions

The present findings suggest that when using CCM Express and CPX Ultima CardiO2 breath-by-breath metabolic carts, both the SSt and filtering methods yield the lowest RMR and RQ estimates with the lowest amount of variability (i.e., lowest intra-measurement coefficients of variation). Moreover, the filtering method might be a valid alternative for use with subjects who do not achieve a steady respiratory state.

## Figures and Tables

**Table 1:** Subject descriptive characteristics.

	Young Adults (*n* = 107)				Middle-Aged Adults (*n* = 74)			
		Min	Max	Percentile 10–90		Min	Max	Percentile 10–90
Age (years)	22.2 ± 2.2	18.2	26.6	19.1–25.2	53.5 ± 5.3	45.0	66.0	47.0–61.7
Sex (*n* %)								
Women	72, 67				39, 53 35, 47			
Men	35, 33							
Metabolic cart used (*n* %)								
CCM Express	46, 43 61, 57 69.3 ± 15.9 167.8 ± 8.7 24.5 ± 4.4 79.8 ± 13.2 41.4 ± 9.5 24.0 ± 8.9 35.0 ± 7.8				0, 0			
CPX Ultima CardiO2					74, 100			
Body weight (kg)		45.0	118.5	52.2–90.6	75.7 ± 15.0 167.8 ± 9.8 26.7 ± 3.8 95.1 ± 11.7 43.5 ± 11.7 30.0 ± 8.4 39.9 ± 9.1	50.6	110.7	57.8–94.6
Height (cm)		148.5	195.1	157.2–180		148.3	189.8	155.8–181.6
BMI (kg/m^2^)		17.2	38.4	19.4–30.9		19.0	38.0	22.0–31.7
Waist circumference (cm)		58.0	125.6	65.0–97.8		68.6	118.7	79.2–107.8
Lean mass (kg)		28.1	66.8	31.0–55.2		22.7	63.6	30.5–59.6
Fat mass (kg)		9.9	51.7	14.6–36.4		14.5	55.8	20.6–40.7
Fat mass (%)		15.3	51.9	26.2–44.4		23.0	59.4	26.7–51.1

Data are presented as mean ± SD unless otherwise stated.

**Table 2:** Variance in resting metabolic rate (RMR) explained by sex and body weight in each of the gas exchange data selection methods.

Method of Data Selection	Young Adults (*n* = 107)						Middle-Aged Adults (*n* = 74)					
			Sex		Weight (kg)				Sex		Weight (kg)	
	Model R^2^	Constant	β	*p*	β	*p*	Model R^2^	Constant	β	*p*	β	*p*
TI 6–10 min	0.44	1232	−171.6	**0.001**	7.8	**<0.001**	0.67	1503	−424.0	**0.001**	8.9	**0.001**
TI 11–15 min	0.40	1340	−214.4	**<0.001**	7.3	**<0.001**	0.67	1616	−418.5	**0.001**	7.1	**0.004**
TI 16–20 min	0.40	1300	−214.5	**<0.001**	7.6	**<0.001**	0.60	1758	−445.2	**0.001**	6.2	**0.031**
TI 21–25 min	0.36	1342	−199.5	**0.001**	6.7	**<0.001**	0.68	1935	−507.5	**0.001**	4.9	0.051
TI 26–30 min	0.34	1208	−155.7	**0.009**	7.5	**<0.001**	0.54	1754	−409.8	**0.001**	5.5	0.058
TI 6–30 min	0.42	1284	−191.2	**0.001**	7.4	**<0.001**	0.66	1713	−441.0	**0.001**	6.5	**0.010**
TI 6–25 min	0.43	1303	−200.0	**<0.001**	7.3	**<0.001**	0.67	1703	−448.8	**0.001**	6.8	**0.008**
SSt 3 min	0.39	1245	−194.9	**0.001**	7.8	**<0.001**	0.66	1669	−456.7	**0.001**	7.1	**0.008**
SSt 4 min	0.39	1169	−167.5	**0.004**	7.9	**<0.001**	0.67	1786	−481.1	**0.001**	6.4	**0.016**
SSt 5 min	0.36	1301	−207.0	**0.001**	7.1	**<0.001**	0.66	1763	−464.1	**0.001**	6.2	**0.016**
SSt 10 min	0.41	1232	−182.8	**0.001**	7.7	**<0.001**	0.66	1681	−443.8	**0.001**	7.0	**0.008**
Low-filter	0.45	1188	−170.6	**0.001**	8.1	**<0.001**	0.67	1701	−442.9	**0.001**	6.6	**0.009**
Medium-filter	0.43	1209	−170.8	**0.001**	7.8	**<0.001**	0.68	1725	−447.5	**0.001**	6.4	**0.010**
Strong-filter	0.44	1218	−182.0	**0.001**	7.7	**<0.001**	0.67	1684	−431.7	**0.001**	6.2	**0.012**

Unstandardized beta and *p*-values (significant values in bold) from multiple regression analyses, in which sex and body weight were included as independent variables, and the RMR estimates yielded by the different methods of gas exchange data selection were included as dependent variables. Sex: 1 = men; 2 = women.

**Table 3:** Variance in resting metabolic rate (RMR) explained by sex, lean mass (LM) and fat mass (FM) in each of the gas exchange data selection methods.

Method of Data Selection	Young Adults (*n* = 107)								Middle-Aged Adults (*n* = 74)							
			Sex		LM (kg)		FM (kg)				Sex		LM (kg)		FM (kg)	
	Model R^2^	Constant	β	*p*	β	*p*	β	*p*	Model R^2^	Constant	β	*p*	β	*p*	β	*p*
TI 6–10 min	0.47	668	−3.5	0.967	18.7	**<0.001**	2.1	0.450	0.66	1436	−400.4	**0.001**	10.5	**0.020**	8.4	**0.011**
TI 11–15 min	0.45	483	39.2	0.683	23.4	**<0.001**	−1.2	0.697	0.67	1583	−407.0	**0.001**	8.0	**0.050**	6.8	**0.022**
TI 16–20 min	0.44	497	23.2	0.814	22.8	**<0.001**	−0.3	0.925	0.60	1533	−370.0	**0.001**	10.4	**0.029**	4.3	0.210
TI 21–25 min	0.41	542	37.0	0.702	21.7	**<0.001**	−1.2	0.697	0.68	1713	−433.1	**0.001**	9.0	**0.030**	3.0	0.320
TI 26–30 min	0.38	433	73.8	0.451	22.1	**<0.001**	−0.2	0.957	0.55	1500	−324.4	**0.005**	10.2	**0.034**	3.3	0.346
TI 6–30 min	0.47	525	34.0	0.701	21.7	**<0.001**	−0.2	0.955	0.66	1553	−381.0	**0.001**	9.6	**0.022**	5.2	0.089
TI 6–25 min	0.47	548	24.0	0.787	21.6	**<0.001**	−0.2	0.956	0.67	1566	−402.5	**0.001**	9.5	**0.024**	5.6	0.065
SSt 3 min	0.44	417	49.5	0.610	23.3	**<0.001**	−0.3	0.919	0.66	1657	−452.0	**0.001**	7.6	0.085	7.0	**0.029**
SSt 4 min	0.45	306	87.7	0.344	24.2	**<0.001**	−0.5	0.868	0.67	1695	−451.0	**0.001**	8.2	0.060	5.7	0.073
SSt 5 min	0.42	379	65.7	0.510	24.3	**<0.001**	−2.0	0.530	0.66	1694	−441.0	**0.001**	7.7	0.073	5.7	0.066
SSt 10 min	0.45	464	44.8	0.628	22.2	**<0.001**	0.1	0.965	0.66	1534	−394.1	**0.001**	9.9	**0.023**	5.7	0.069
Low-filter	0.50	451	47.8	0.571	22.1	**<0.001**	0.8	0.770	0.67	1577	−400.9	**0.001**	9.1	**0.030**	5.6	0.066
Medium-filter	0.48	424	62.0	0.471	22.7	**<0.001**	0.1	0.989	0.67	1597	−404.1	**0.001**	8.9	**0.029**	5.3	0.073
Strong-filter	0.49	416	55.4	0.516	22.8	**<0.001**	−0.3	0.918	0.67	1534	−381.2	**0.001**	9.1	**0.025**	4.9	0.094

Unstandardized beta and *p*-values (significant values in bold) from multiple regression analyses, in which sex, LM and FM were included as independent variables, and the RMR estimates yielded by the different methods of gas exchange data selection were included as dependent variables. Sex: 1 = men; 2 = women.

## Supplementary Materials

The following data are available online at https://www.mdpi.com/2072-6643/12/2/487/s1: Figure S1: Differences among methods for gas exchange data selection with respect to VO_2_ and VCO_2_, Figure S2: Period from the 30 min RMR measurement in which the SSt is achieved, Figure S3: Differences among 5 min SSt method achieved at different time lengths gas exchange data selection with respect to RMR, VO_2_, VCO_2_, RQ, and Mean All CVs, Figures S4 and S5: Differences among gas exchange data selection with respect to the CV VO_2_, CV VCO_2_, CV RQ, CV VE, and the Mean All CVs, and Table S1: post-hoc Bonferroni analysis.
