# Supplementary material for: Impact of the Method Used to Select Gas Exchange Data for Estimating the Resting Metabolic Rate, as Supplied by Breath-by-Breath Metabolic Carts
Source: Nutrients. 2020 Feb 14;12(2):487. doi: 10.3390/nu12020487 (PMC7071290; doi:10.3390/nu12020487)
Supplement: Supplementary file 1 [file nutrients-12-00487-s001.pdf]

Table S1. Differences in resting metabolic rate (RMR), respiratory quotient (RQ), fat oxidation (FATOx), carbohydrate oxidation (CHOOx), oxygen consumption (VO_2) and carbon dioxide production (VCO_2) across methods for gas exchange data analysis, as revealed by *post-hoc* Bonferroni analysis.

		Middle-aged adults (n=74)													
	RMR (kcal/day)	TI-6-10 min	TI-11-15 min	TI-16-20 min	TI-21-25 min	TI-26-30 min	TI-6-30 min	TI-6-25 min	SSt-3 min	SSt-4 min	SSt-5 min	SSt-10 min	Low filter	Medium filter	Strong filter
Young adults (n=107)	TI-6-10 min														**0.019**
	TI-11-15 min														
	TI-16-20 min														**<0.001**
	TI-21-25 min														**0.004**
	TI-26-30 min														**0.009**
	TI-6-30 min														**<0.001**
	TI-6-25 min														**<0.001**
	SSt-3 min														
	SSt-4 min	**0.023**	**0.01**				**0.001**	**0.001**							
	SSt-5 min		**<0.001**				**0.005**	**0.001**							
	SSt-10 min							**0.021**							**<0.001**
	Low filter														**<0.001**
	Medium filter									**0.019**					**<0.001**
	Strong filter	**0.001**	**<0.001**				**<0.001**	**<0.001**					**<0.001**	**<0.001**	

		Middle-aged adults (n=74)													
	RQ (VCO_2/VO_2)	TI-6-10 min	TI-11-15 min	TI-16-20 min	TI-21-25 min	TI-26-30 min	TI-6-30 min	TI-6-25 min	SSt-3 min	SSt-4 min	SSt-5 min	SSt-10 min	Low filter	Medium filter	Strong filter
Young adults (n=107)	TI-6-10 min														
	TI-11-15 min														
	TI-16-20 min														
	TI-21-25 min														
	TI-26-30 min														
	TI-6-30 min					**0.046**									
	TI-6-25 min					**0.030**	**0.019**								
	SSt-3 min					**<0.001**									
	SSt-4 min					**<0.001**									
	SSt-5 min					**0.001**									
	SSt-10 min					**0.003**									
	Low filter					**0.002**	**0.006**								
	Medium filter					**0.004**	**0.030**								
	Strong filter					**0.001**	**0.007**								

		Middle-aged adults (n=74)													
	FATOx (g/min)	TI-6-10 min	TI-11-15 min	TI-16-20 min	TI-21-25 min	TI-26-30 min	TI-6-30 min	TI-6-25 min	SSt-3 min	SSt-4 min	SSt-5 min	SSt-10 min	Low filter	Medium filter	Strong filter
Young adults (n=107)	TI-6-10 min														
	TI-11-15 min														
	TI-16-20 min														
	TI-21-25 min														
	TI-26-30 min														
	TI-6-30 min														
	TI-6-25 min														
	SSt-3 min					**0.007**									
	SSt-4 min					**0.005**									
	SSt-5 min					**0.019**									

SSt-10 min					**0.005**									
Low filter					**0.006**									
Medium filter					**0.008**									
Strong filter					**0.002**									

Middle-aged adults (n=74)

Young adults (n=107)

CHOOx (g/min)	TI-6-10 min	TI-11-15 min	TI-16-20 min	TI-21-25 min	TI-26-30 min	TI-6-30 min	TI-6-25 min	SSt-3 min	SSt-4 min	SSt-5 min	SSt-10 min	Low filter	Medium filter	Strong filter
TI-6-10 min														
TI-11-15 min														
TI-16-20 min														
TI-21-25 min														
TI-26-30 min														
TI-6-30 min														
TI-6-25 min														
SSt-3 min					**0.001**									
SSt-4 min					**<0.001**	**0.003**								
SSt-5 min					**0.001**	**0.025**								
SSt-10 min					**0.002**	**0.036**								
Low filter					**<0.001**	**<0.001**	**0.032**							
Medium filter					**0.001**	**0.001**								
Strong filter				**0.005**	**<0.001**	**<0.001**	**0.001**							

Middle-aged adults (n=74)

Young adults (n=107)

VO_2 (ml/min)	TI-6-10 min	TI-11-15 min	TI-16-20 min	TI-21-25 min	TI-26-30 min	TI-6-30 min	TI-6-25 min	SSt-3 min	SSt-4 min	SSt-5 min	SSt-10 min	Low filter	Medium filter	Strong filter
TI-6-10 min														**0.02**
TI-11-15 min														
TI-16-20 min														**<0.001**
TI-21-25 min														**0.012**
TI-26-30 min														**0.026**
TI-6-30 min														**<0.001**
TI-6-25 min														**<0.001**
SSt-3 min														
SSt-4 min	**0.038**	**0.044**				**0.006**	**0.004**							
SSt-5 min		**<0.001**				**0.044**	**0.004**							
SSt-10 min							**0.042**							**<0.001**
Low filter														**<0.001**
Medium filter									**0.041**					**<0.001**
Strong filter	**0.003**	**<0.001**				**<0.001**	**<0.001**					**<0.001**	**<0.001**	

Middle-aged adults (n=74)

Young adults (n=107)

VCO_2 (ml/min)	TI-6-10 min	TI-11-15 min	TI-16-20 min	TI-21-25 min	TI-26-30 min	TI-6-30 min	TI-6-25 min	SSt-3 min	SSt-4 min	SSt-5 min	SSt-10 min	Low filter	Medium filter	Strong filter
TI-6-10 min														
TI-11-15 min														
TI-16-20 min														**0.003**
TI-21-25 min														**0.02**
TI-26-30 min														**0.002**
TI-6-30 min														**<0.001**
TI-6-25 min														**<0.001**
SSt-3 min														

SSt-4 min	**0.046**	**0.001**		**0.005**	**0.001**	**<0.001**	**<0.001**							
SSt-5 min		**0.002**			**0.019**	**<0.001**	**<0.001**							
SSt-10 min						**0.008**	**0.036**							**<0.001**
Low filter						**<0.001**	**0.004**		**0.043**					**<0.001**
Medium filter						**<0.001**	**0.01**		**0.02**					**<0.001**
Strong filter	**<0.001**	**<0.001**	**0.015**	**<0.001**	**<0.001**	**<0.001**	**<0.001**				**0.005**	**<0.001**	**<0.001**	

Significant P values returned by the post-hoc Bonferroni test are presented. P values for young adults are presented shaded in white while P values for middle-aged adults are presented shaded in light grey.

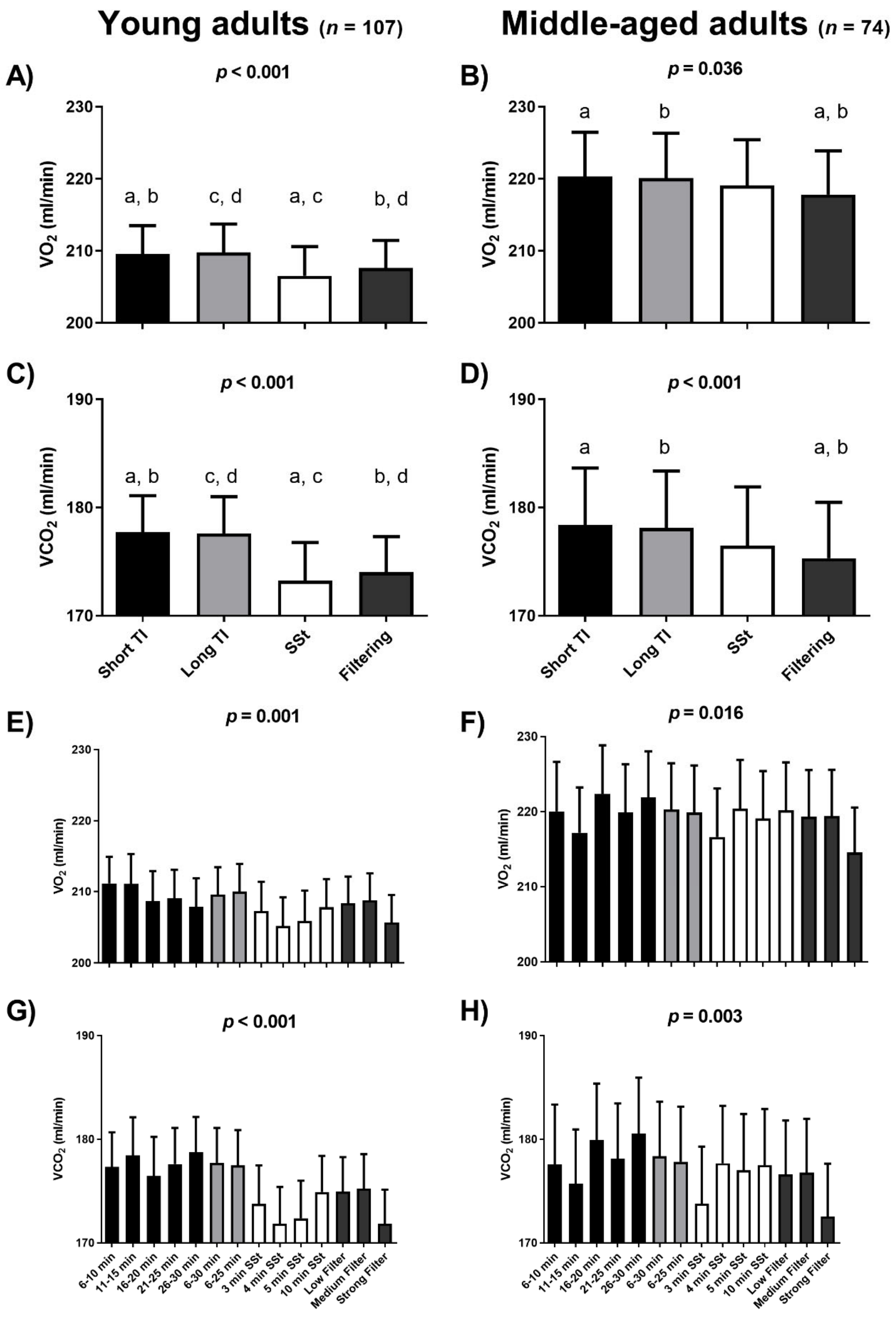

Figure S1. Differences among methods for gas exchange data selection with respect to oxygen consumption (VO_2) and carbon dioxide production (VCO_2). Black columns represent short time interval (TI) periods (i.e. the means of the per minute ventilation data [pMVD] values for all variables available for these time

periods, Panels A-D; the pMVD values for each short TI period, Panels E-F); light grey columns represent long TI periods (i.e. the means of the pMVD values for all variables available for these time periods, Panels A-D; the means of the pMVD values for each long TI period, Panels E-F); white columns represent steady state (SSt) periods (i.e. the means of the pMVD values for all variables available for these SSt periods, Panels A-D; the means of the pMVD values for each SSt period, Panels E-F); dark grey columns represent filtering methods (i.e. the means of the pMVD values for all variables available for these filtering periods, Panels A-D; the means of the pMVD values for each filtering period, Panels E-F). P values come from repeated-measures analysis of variance (ANOVA). Identical indicatory letters highlight differences as determined by post-hoc Bonferroni analysis. Data are presented as mean and standard error of the mean (SEM). Mil: millilitres; Min: minutes.

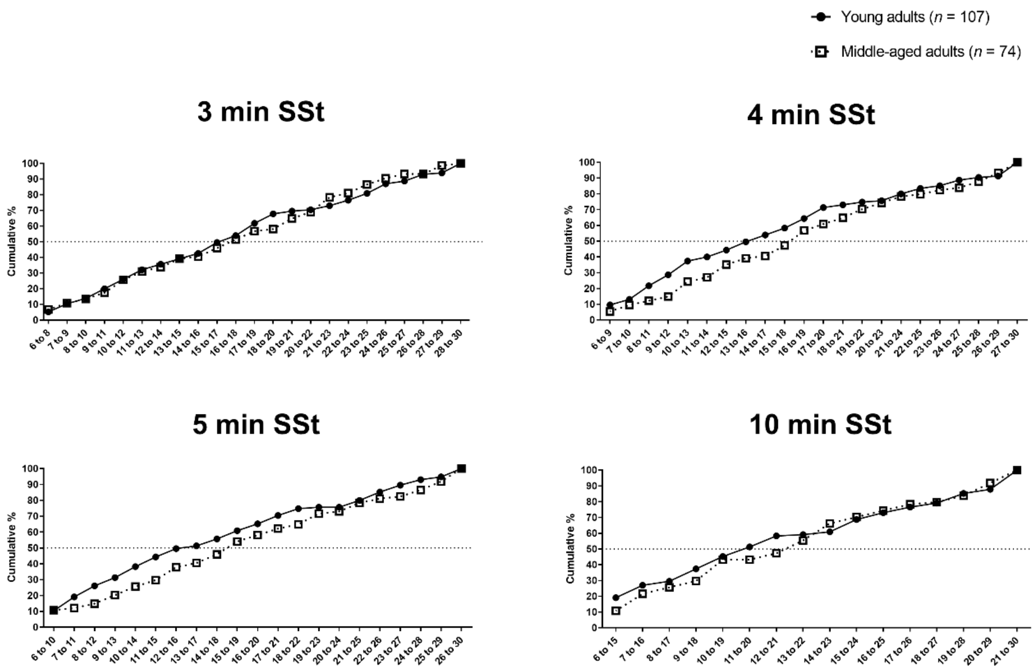

Figure S2. Period from the 30 min resting metabolic rate measurement in which the steady state (SSt) is achieved with respect to the SSt methods applied in each cohort. Data are presented as a cumulative percentage of subjects whom achieved the SSt. Solid line represent the percentage for the young-adults' cohort; dashed line represent the percentage for the middle-aged adults' cohort.

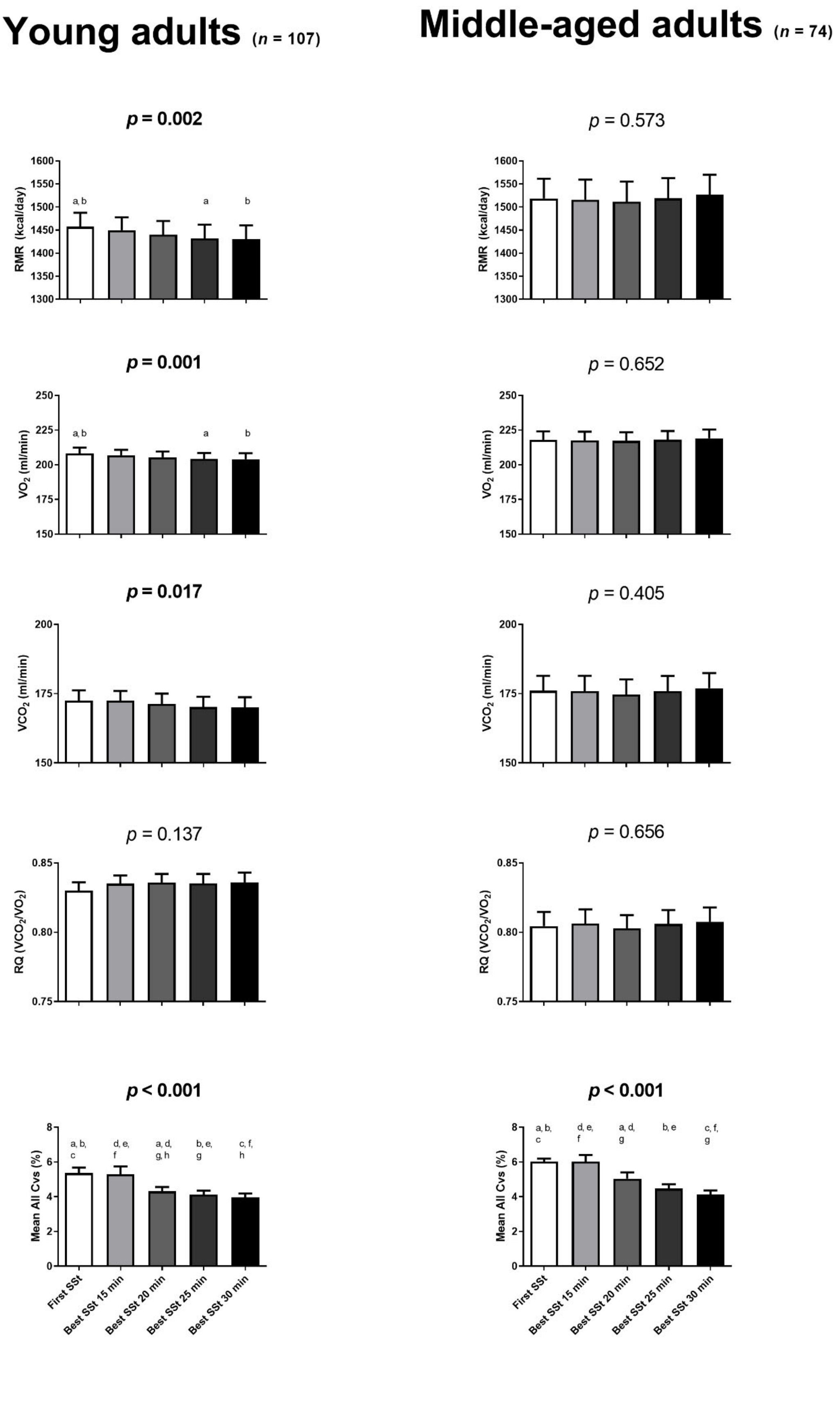
Young adults (n = 107)
Middle-aged adults (n = 74)
p = 0.002
RMR (kcal/day)
a, b
a
b
p = 0.573
p = 0.001
VO2 (ml/min)
a, b
a
b
p = 0.652
p = 0.017
VCO2 (ml/min)
p = 0.405
p = 0.137
RQ (VCO2/VO2)
p = 0.656
p < 0.001
Mean All Cvs (%)
a, b, c
d, e, f
a, d, g, h
b, e, g
c, f, h
p < 0.001
a, b, c
d, e, f
a, d, g
b, e
c, f, g
First SSt
Best SSt 15 min
Best SSt 20 min
Best SSt 25 min
Best SSt 30 min

Figure S3. Differences among 5 min Steady State time (SSt) method achieved at different time lengths gas exchange data selection with respect to resting metabolic rate (RMR), oxygen consumption (VO_2), carbon dioxide production (VCO_2), respiratory quotient (RQ) and the mean of all coefficients of variation (Mean All CVs; i.e., coefficient of variation of VO_2, VCO_2, RQ and minute ventilation). First SSt represent the first SSt-5 min period in which the coefficient of variation of VO2, VCO2 and minute ventilation were lower than 10% and the coefficient of variation of RQ was lower than 5%. Best SSt represent the SSt-5 min period with the lowest mean of all coefficients of variation aforementioned (i.e., Mean All CVs). P values come from repeated-measures analysis of variance (ANOVA). Identical indicatory letters highlight differences as determined by post-hoc Bonferroni analysis. Data are presented as mean and standard error of the mean (SEM). Mil: millilitres; Min: minutes.

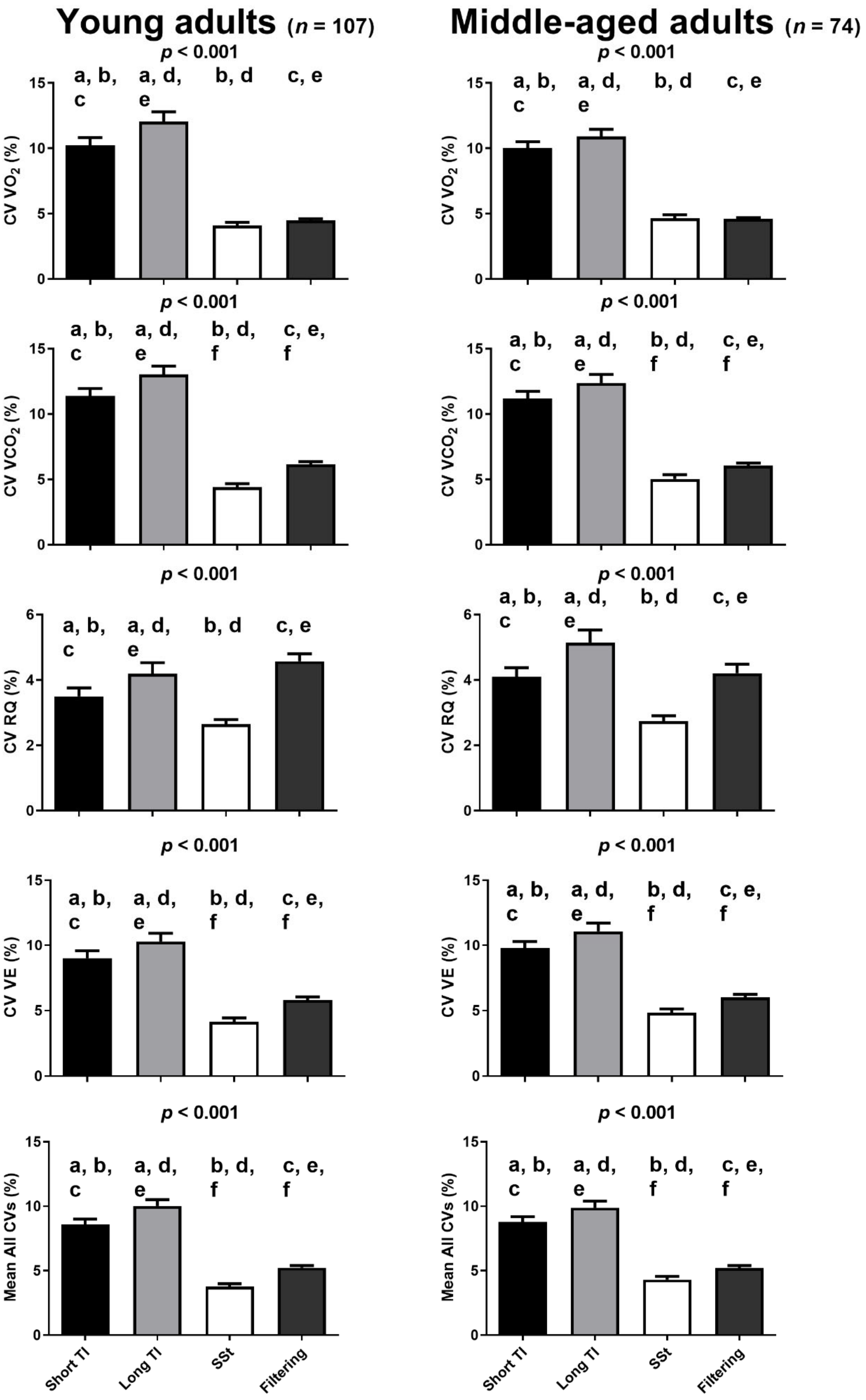
Young adults (n = 107)
Middle-aged adults (n = 74)
p < 0.001
a, b, c
a, d, e
b, d
c, e
b, d, f
c, e, f
CV VO2 (%)
CV VCO2 (%)
CV RQ (%)
CV VE (%)
Mean All CVs (%)
Short TI
Long TI
SSt
Filtering

Figure S4. Differences among gas exchange data selection with respect to the coefficient of variation of oxygen consumption (CV VO_2), carbon dioxide production (CV VCO_2), respiratory quotient (CV RQ), ventilation (CV VE) and the mean of all coefficients of variation aforementioned (Mean All CVs). Black columns represent short time interval (TI) periods; light grey columns represent Whole-measurement periods; white columns represent steady state (SSt) periods; and dark grey columns represent filtering methods. P values come from repeated-measures analysis of variance (ANOVA). Identical indicatory letters highlight differences as determined by post-hoc Bonferroni analysis. Data are presented as mean and standard error of the mean (SEM).

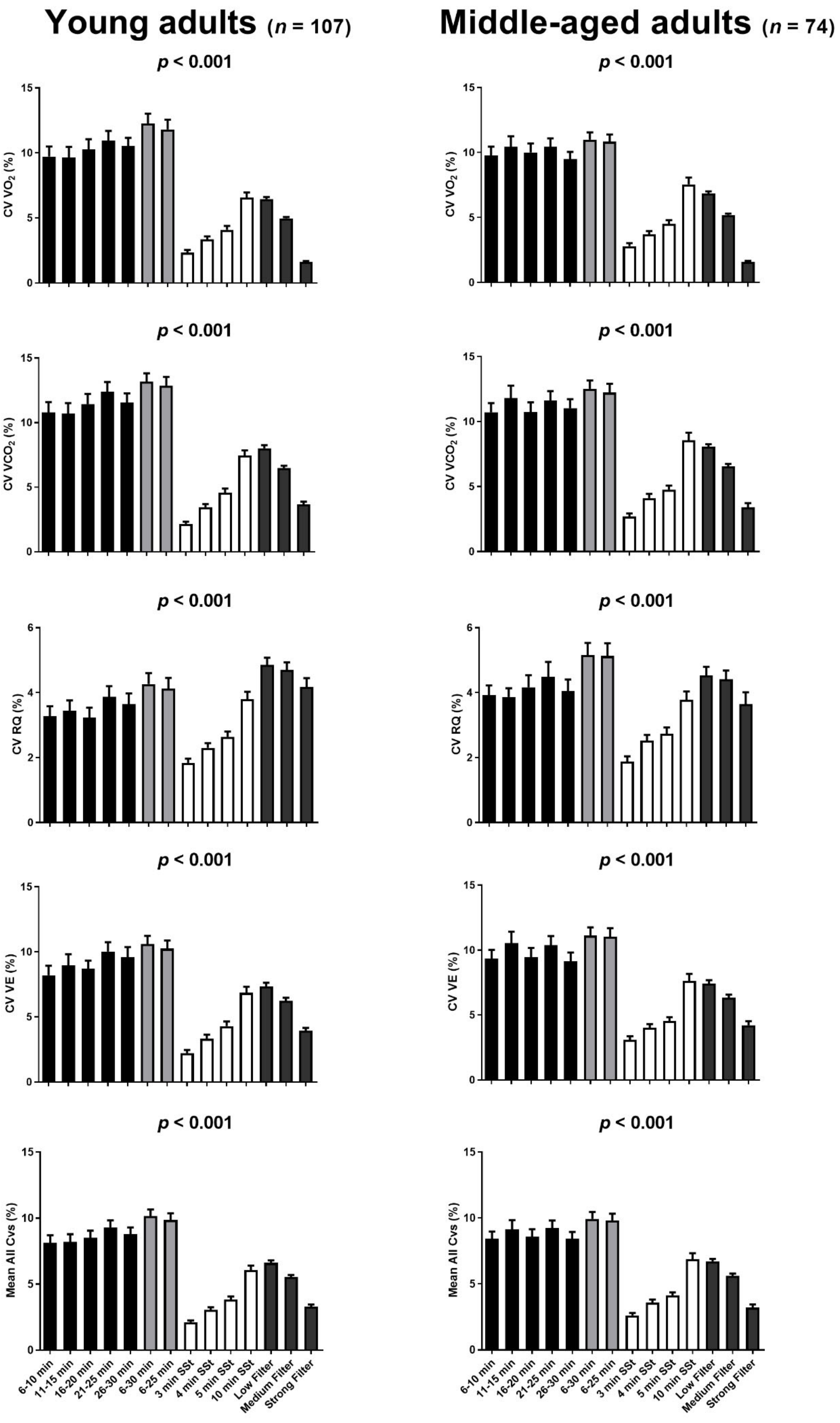
Young adults (n = 107)
Middle-aged adults (n = 74)
p < 0.001
CV VO2 (%)
CV VCO2 (%)
CV RQ (%)
CV VE (%)
Mean All Cvs (%)
6-10 min
11-15 min
16-20 min
21-25 min
26-30 min
6-30 min
6-25 min
3 min SSt
4 min SSt
5 min SSt
10 min SSt
Low Filter
Medium Filter
Strong Filter

Figure S5. Differences among gas exchange data selection methods with respect to coefficient of variation of oxygen consumption (CV VO_2), carbon dioxide production (CV VCO_2), respiratory quotient (CV RQ), ventilation (CV VE), and the mean of all the aforementioned coefficients of variation (Mean All CVs). Black columns represent short time interval (TI) periods; light grey columns represent long TI periods; white columns represent steady state (SSt) periods; dark grey columns represent filtering methods. P values come from repeated-measures analysis of variance (ANOVA). Identical indicatory letters highlight differences as determined by post-hoc Bonferroni analysis. Data are presented as mean and standard error of the mean (SEM).
